# Phytic Acid Doped Polypyrrole as a Mediating Layer Promoting Growth of Prussian Blue on Cotton Fibers for Solar-Driven Interfacial Water Evaporation

**DOI:** 10.3390/polym14010006

**Published:** 2021-12-21

**Authors:** Xueyao Wang, Dongmei Yang, Xianhui An, Xueren Qian

**Affiliations:** Key Laboratory of Bio-Based Material Science & Technology, Ministry of Education, Northeast Forestry University, Harbin 150040, China; 873574352@nefu.edu.cn (X.W.); yyddmm1980122@163.com (D.Y.); anxianh509@163.com (X.A.)

**Keywords:** cotton fibers, Prussian blue, polypyrrole, phytic acid doping, mediating layer, photothermal conversion material

## Abstract

Phytic acid doped polypyrrole (PPy) as a mediating layer was in-situ coated on cotton fibers (CFs) to promote the growth of Prussian blue (PB) and construct the PB/PPy@CFs composite. The results showed that the proper amounts of PA doped PPy in-situ generated significantly promoted the growth of PB on CFs, the PB deposition ratio increased from 12.29% (PB@CFs) to 32.4% (PB/PPy@CFs), and the growth of PB on PPy@CFs could be completed in 4 h. Scanning electron microscopy (SEM) showed that the PB particles with perfect nano cubic structure were formed in the composite. X-ray diffraction (XRD), Fourier transform infrared spectroscopy (FTIR), and X-ray photoelectron spectroscopy (XPS) showed that both PB and PPy were successfully deposited on CFs. The PB/PPy@CFs composite had excellent light absorption, hydrophilicity, wettability, and photothermal property, and the surface could be heated up to 81.5 °C under one sun illumination. The PB/PPy@CFs composite as a photothermal conversion material was used for solar-driven interfacial water evaporation, the water evaporation rate was 1.36 kg·m^−^^2^·h^−^^1^ at the optical concentration of 1 kW·m^2^, and the corresponding photothermal conversion efficiency increased from 81.69% (PB@CFs) to 90.96% (PB/PPy@CFs).

## 1. Introduction

With the great development of the world economy and industry, and people’s pursuit of higher quality of life, the demand for energy is also increasing [1,2]. At the same time, with people’s concern about the increasing depletion of non-renewable fossil fuel resources and the associated environmental problems resulted from their unreasonable utilization, people gradually require their sustainable utilization in the future energy development model. With the background of global warming, “low carbon economy” based on low energy consumption and low pollution has become a global hot spot. Based on this, in recent years, as one of the important innovative ways of green energy utilization, the efficient use of renewable solar energy has become a research hotspot concern for relevant researchers [3,4,5]. Photothermal conversion is an important way to make effective use of solar energy and can expand the application scope of solar energy to relevant fields with practical application needs, such as water environment purification, seawater desalination, photochemical catalysis, and so on [6,7,8,9,10,11].

In the past few years, various materials for solar-driven water evaporation have been prepared and have excellent photothermal efficiency. These materials for photothermal steam generation include plasma materials (e.g., carbon-based materials, metal plasma materials, etc.) and organic materials [12,13]. Among carbon-based materials, graphene, graphene oxide, reduced graphene oxide, carbon nanotubes, carbon particles, and coal-based films have been used as photothermal materials for the generation of solar steam. However, due to the high cost of raw materials, a complex manufacturing process, and other adverse factors, the large-scale application of these materials has low economic benefits.

As a typical transition metal organic framework (MOF), Prussian blue (PB) can be prepared by facile coprecipitation of Fe^3+^ and [Fe(CN)_6_]^4−^ [14]. PB possesses a faced-centered cubic (FCC) crystal structure where alternating Fe^2+^ and Fe^3+^ are bridged by the C≡N (Fe^2+^ bonds with C, Fe^3+^ bonds with N), and such a structure renders a good photostability of PB nanoparticles [15,16]. PB has strong light absorption ability, good thermal stability, high mechanical strength, and low manufacturing cost. It has been demonstrated that PB has excellent photothermal effect arising from a metal-to-metal charge transfer from Fe^2+^ to Fe^3+^ [17,18]. However, PB is a powder material and not easy to form, which greatly limits its application.

Cellulose is widely used in various fields as a renewable and biodegradable material with rich sources and low cost. Cellulose is easy to form and process and is a suitable carrier for PB. In the work from Fang et al., PB nanocrystals were first synthesized and in-situ loaded on cotton fibers (CFs) to form a photothermal composite material for solar-driven organic solvent purification [19]. Their results showed that the amount of PB loaded on CFs could reach 16.7 g·m^−2^ (i.e., 11.9%), and the PB@CFs surface could be heated up to 71 °C under one sun illumination. However, we believe that there is room for further improvement in the loading amount of PB on CFs and the photothermal properties of the composite. Polypyrrole (PPy), one of the conductive polymers, has the advantages of easy synthesis, good air stability, chemical doping, and excellent photothermal properties, and can absorb near-infrared light and convert it into heat energy [20]. Phytic acid (PA) as a dopant of conductive polymers is conducive to the adsorption of metal ions [21,22].

**Scheme 1:**
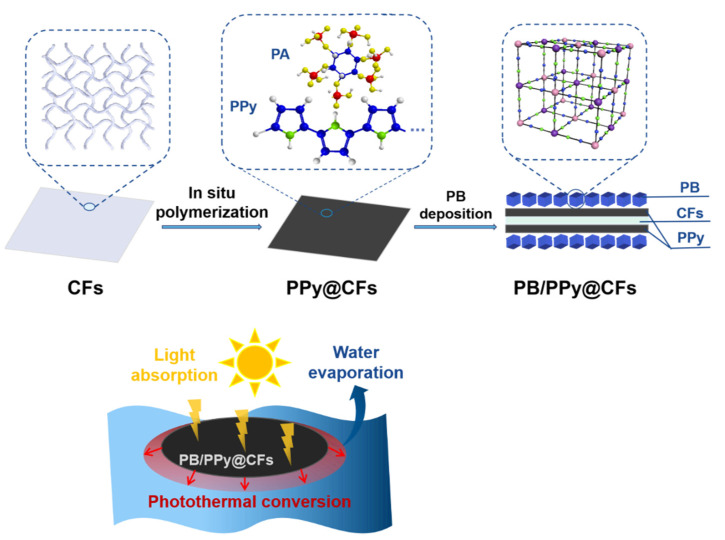
Schematic illustration of preparation and photothermal interfacial water evaporation application of PB/PPy@CFs composite.

In this work, we used phytic acid (PA) doped PPy as a mediating layer to promote the growth of PB on CFs utilizing the electrostatic adsorption of amino groups in PPy on Fe^3+^ and the chelation of phosphate groups in PA, and the PB/PPy@CFs composite was obtained and used as a photothermal conversion material for solar-driven interfacial water evaporation (Scheme 1).

## 2. Experiment

### 2.1. Materials and Reagents

Cotton fibers (i.e., cotton cloth, abbreviated as CFs) used in this experiment were provided by Yongsheng cotton mill (Jinzhou, China). Before use, they were boiled in boiling water for 4 h and dried at 60 °C. Polyethylene foam was purchased from Jimeilin Materials Co., Ltd. (Langfang, China). Pyrrole (Py, analytical purity) and phytic acid (PA, ≥70%) were produced by Sinopharm Chemical Reagent Co., Ltd. (Shanghai, China). Copper sulfate pentahydrate (98%) and potassium ferricyanide (≥99.5%) were produced by Aladdin reagent (Shanghai) Co., Ltd. Ammonia (25–28%) and ferric chloride hexahydrate (analytical purity) were produced by McLean Reagent Co., Ltd. (Shanghai, China). Hydrochloric acid (36–38%) was produced by Xilong Technology Co., Ltd. (Shantou, China). Deionized water was made by our laboratory. Except for that the Py monomer was distilled under reduced pressure before use, all chemicals were directly used without further purification.

### 2.2. Preparation of CFs-Based Composites

#### 2.2.1. Preparation of PPy@CFs Composite

Polypyrrole@cotton fibers (PPy@CFs) composite was prepared by in-situ polymerization process. Typically, 2 g of oven-dry cotton cloth (2 cm × 2 cm) was placed in 160 mL deionized water and 3 mmol of PA was added. Then, 9 mmol of pyrrole (Py) dissolved in 10 mL of absolute ethanol was added to the above mixture system. Next, 2.43 g of ferric chloride hexahydrate (FeCl_3_·6H_2_O) dissolved in 30 mL of deionized water was added to the system and stirred continuously at 0–5 °C for 2 h (the molar ratio of FeCl_3_ to Py was 1:1). Finally, PA doped PPy@CFs composite was obtained by washing and drying at 60 °C.

#### 2.2.2. Preparation of PB@CFs Composite

Prussian blue@cotton fibers (PB@CFs) composite was prepared by simple in-situ growth method. Typically, 2 g of dried cotton cloth was immersed in 150 mL of deionized water containing 1.5 mol of potassium ferricyanide for 4 h. Then, 1.5 mol of ferric chloride dissolved in 150 mL of deionized water was added and stirred magnetically for 4 h. Finally, PB@CFs composite was obtained by washing and drying at 60 °C.

#### 2.2.3. Preparation of PB/PPy@CFs Composite

Typically, 2 g of cotton cloth with a thickness of 0.381 mm was added into 160 mL deionized water and added 3 mmol of PA. Then, 9 mmol of Py dissolved in 10 mL of absolute ethanol was added to the above mixture system. Next, 2.43 g of ferric chloride hexahydrate (FeCl_3_·6H_2_O) dissolved in 30 mL of deionized water was added to the system and stirred continuously at 0–5 °C for 2 h. PPy@CFs was obtained by washing with deionized water, and then, 2 g of dried PPy@CFs was immersed in 150 mL of deionized water containing 1.5 mol of potassium ferricyanide for 4 h. Then, 1.5 mol of ferric chloride dissolved in 150 mL of deionized water was added and stirred magnetically for 4 h. Finally, PB/PPy@CFs with a thickness of 0.42 mm was obtained. The thickness of solar absorber layer can be calculated as 19.5 μm. The thickness of the samples was measured using an IMT-HD02 thickness tester. In this work, the effects of Py dosage (0–13 mmol), PA dosage (0–3.5 mmol), and PB growth time (2–6 h) on PB deposition were investigated. Unless otherwise stated, other process conditions remained unchanged.

### 2.3. Calculation of PB Deposition Ratio

The as-prepared product was dried in an oven at 105 °C for 6 h. After cooling for 30 min in a dryer, the mass was measured. The deposition ratio (*D*, %) of PB was calculated according to the following formula:

*D* = (*M*_2_ − *M*_1_)/*M*_0_ × 100% (1)

where, *M*_0_ is the original mass of CFs, g; *M*_1_ is the mass of the composite coated PPy layer, g; *M*_2_ is the mass of the composite deposited PB, g.

### 2.4. Solar-Driven Water Evaporation Experiment and Characterization

#### 2.4.1. Wettability

The hydrophilic properties of samples were tested and characterized by DSA30 water contact angle analyzer produced by Kruss Co., Ltd. (Hamburg, Germany).

The wettability of samples was tested by strip wicking method. The upper ends of two cloth strips (2 cm × 8 cm) were fixed on the iron wire with clips, the lower end of one cloth strip was contacted with water, and the lower end of the other cloth strip was exposed to air. After 3 min, they were photographed by FLIR A35 FOV 45 infrared thermal imager to observe the wetting situation of samples.

#### 2.4.2. Photothermal Performance

CEL-S500/350 xenon lamp was used to simulate solar radiation to study the solar thermal characteristics of samples, and FLIR A35 FOV 45 infrared thermal imager was used to monitor the surface temperature under solar irradiation.

#### 2.4.3. Solar-Driven Interface Water Evaporation

**Figure 1:**
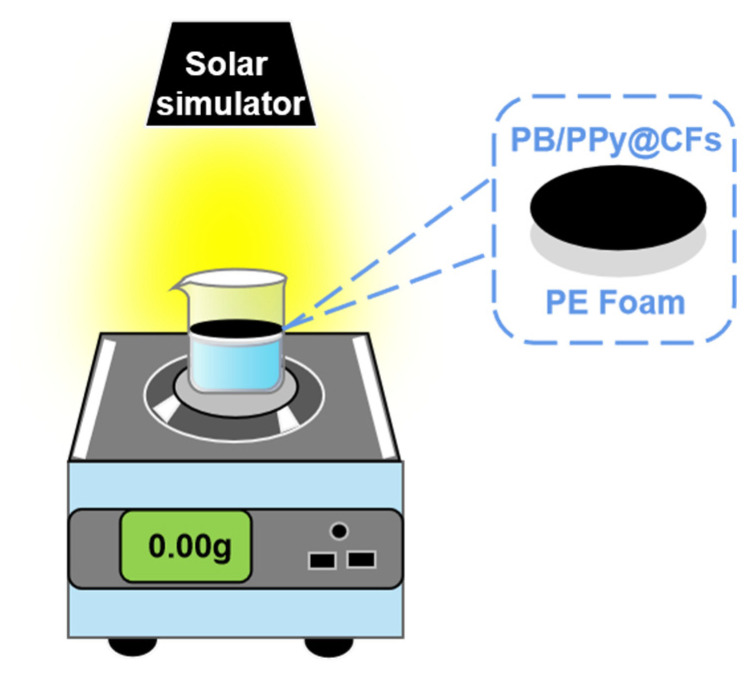
Solar-driven interface water evaporation test device.

The solar-driven interface water evaporation test device is shown in Figure 1. The experiments were conducted under laboratory conditions (ambient temperature ~25 °C and ~40% relative humidity). The water evaporation performance of each sample was studied using xenon lamp as the illuminator, equipped with an optical filter. Weight changes were monitored using an electronic analytical scale (accurate to 0.1 mg) and were recorded in real-time. The circular sample with a diameter of 2 cm was supported by a polyethylene foam with the same size to prevent the water-absorbed sample from sinking to the bottom and precisely located at the gas-liquid interface of water. In order to better compare the water evaporation performance of different samples, the xenon lamp for water evaporation experiment was turned on after the sample was wetted for 10 min. The distance between xenon lamp and solar absorbers was determined based on an optical power meter.

The evaporation rate was calculated by the following formula [23]:

*V* = (*M*_1_ − *M*_2_)/(*A·*∆*t*) (2)

where, *V* is the evaporation rate, kg·m^−2^·h^−1^; *M*_1_ is the initial mass of the liquid, kg; *M*_2_ is the mass of the liquid under different illumination time, kg; *A* is the area of the tested sample, m^2^; ∆*t* is the time interval recording the liquid mass, h.

In the study of the materials for interfacial water evaporation, the performance of materials is often evaluated by photothermal conversion efficiency (i.e., the proportion of the energy consumed in the water evaporation in the total energy input). There are two kinds of photothermal conversion efficiency, i.e., those calculated with and without the effect of water evaporation in the dark [24,25]. We chose the latter to calculate the photothermal conversion efficiency (*η*), i.e., *η* was calculated in the absence of the effect of water evaporation in the dark according to the following formula [26]:

*η* = (*V*·*h*_LV_)/(*C*_opt_·*P*_0_) (3)

where, *η* is the photothermal conversion efficiency, %); *V* is the evaporation rate, kg·m^−2^·h^−1^, *h*_LV_ is the total evaporation enthalpy (i.e., the sum of sensible heat and latent heat) in water evaporation experiment, kJ·kg^−1^; *C*_opt_ is the optical concentration of simulated solar light source, kW·m^−2^; *P*_0_ is the direct solar irradiation intensity, i.e., 1 kW·m^−2^.

#### 2.4.4. Stability

The water evaporation system was placed at different optical concentrations to obtain the water evaporation rate after continuous irradiation. The optical concentration increased first and then decreased. The water evaporation system was irradiated continuously for 2 h at each optical concentration.

### 2.5. SEM, XRD, FTIR, XPS and UV-Vis-NIR Spectroscopy Characterization

Zeiss sigma 300 scanning electron microscope (Carl Zeiss AG, Oberkochen, Germany) with intelligent EDX spectrometer was used to analyze the morphology of samples at an accelerating voltage of 0.02–30 kV. Before observation, the sample surface was coated with gold under vacuum. RIGAKU Ultima IV X-ray diffractometer (Beijing Guanyuan Technology Co., Ltd., Beijing, China) was used to analyze the crystalline nature of samples. The wavelength of the nickel-filtered Cu-Kα radiation source was 1.5418 Å, the voltage is 40 kV, and the current is 40 mA. Thermo Scientific Nicolet iS5 FT-IR spectrometer (Thermo Fisher Scientific, Waltham, MA, USA) was used to record the FTIR spectra of samples in the frequency range of 4000–400 cm^−1^. ESCALAB 250xi X-ray photoelectron spectrometer with Al-Kα radiation source (*hv* = 1486.6 eV) was used to measure the element composition and valence information of samples. UV-3600i Plus UV-Vis-NIR spectrophotometer (Shimadzu International Trade Co., Ltd., Shanghai, China) was used to record the absorption spectra of samples in a wavelength range of 200–2500 nm.

## 3. Results and Discussion

### 3.1. Promoting Effect of PPy on the Growth of PB

**Figure 2:**
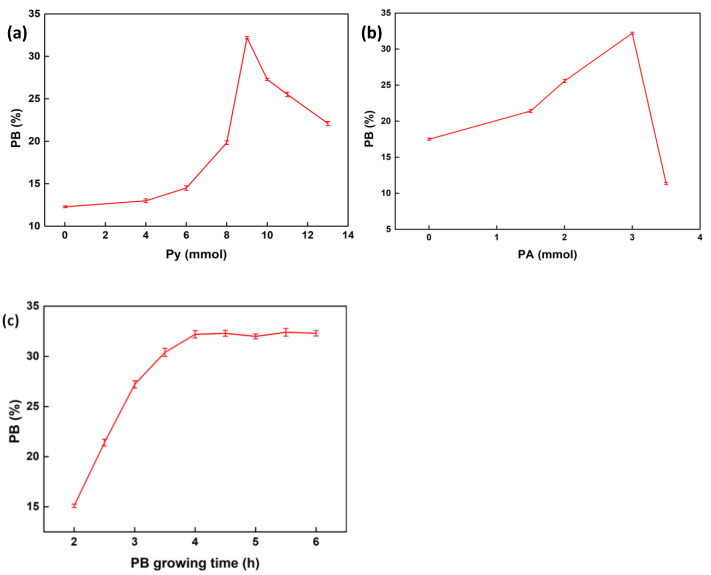
Effect of Py dosage (**a**), PA dosage (**b**), and PB growing time (**c**) on PB deposition.

Figure 2a,b shows the variation of PB deposition ratio with Py and PA dosage. The PB deposition ratio was only 12.29% when PB was directly grown on CFs without PPy mediation. As shown in Figure 2a, PB deposition ratio first increased and then decreased with Py dosage, its value was the highest (32.4%) when the Py dosage was 9 mmol, indicating that an appropriate amount of PPy coated as a mediating layer can promote the growth of PB on CFs. The PB deposition ratio was 17.4% when no PA was added (Figure 2b). With the increase of PA dosage, the PB deposition ratio gradually increased and reached the highest (32.4%) when the PA dosage was 3 mmol. It might be that PA doped PPy also had the chelation effect between the phosphate group in PA and Fe^3+^ in PB, so the in-situ growth of PB was further improved. The deposition ratio of PB decreased when PA dosage was beyond 3 mmol, which might be because too many PA molecules occupied the active sites of PPy.

Figure 2c shows the variation of deposition ratio of PB with growing time. The deposition ratio of PB increased with the increase of growing time and reached the maximum at 4 h. After that, the deposition ratio of PB did not increase again. This result indicated that the growth of PB on CFs was a process with relatively moderate and industrially acceptable speed, and could be completed in 4 h.

### 3.2. Morphology and Structure of Composites

#### 3.2.1. SEM Observation

**Figure 3:**
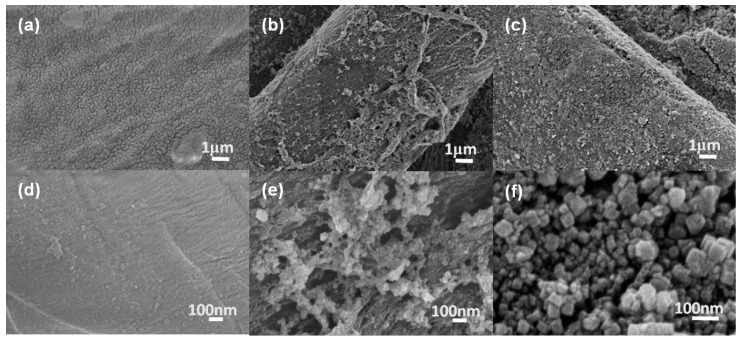
SEM images of CFs (**a**,**d**), PPy@CFs (**b**,**e**), and PB/PPy@CFs (**c**,**f**) at 20 kx and 150 kx magnifications.

Figure 3 shows SEM images of CFs, PPy@CFs and PB/PPy@CFs at 20 kx and 150 kx magnifications. The untreated CFs had rich pore structure (Figure 3a,d), which was conducive to the growth of active substances. As shown in Figure 3b,e, PPy had been successfully coated on the surface of CFs. FeFe(CN)_6_ nanoparticles were uniformly grown on the surface of PPy@CFs (Figure 3c). The perfect nano cubic structure of FeFe(CN)_6_ particles was observed from Figure 3f, indicating that there was a strong binding force between PPy and FeFe(CN)_6_, and PPy mediating layer greatly promoted PB growth on CFs.

#### 3.2.2. XRD Analysis

**Figure 4:**
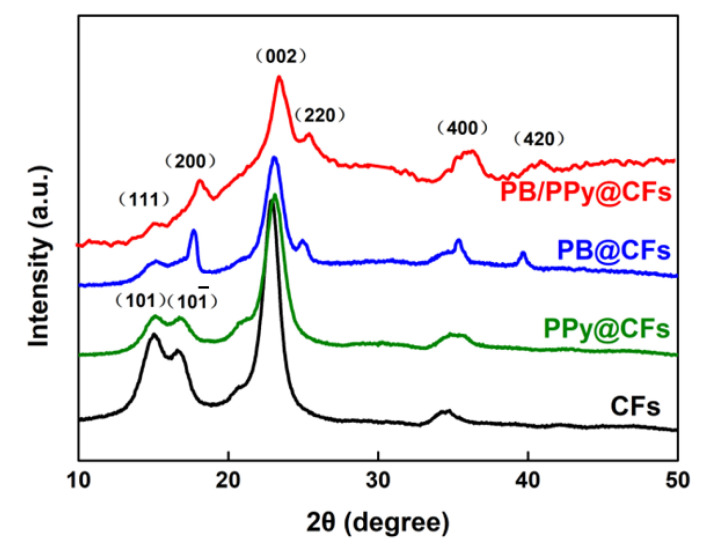
XRD curves of CFs, PPy@CFs, PB@CFs, and PB/PPy@CFs.

Figure 4 shows XRD patterns of CFs, PPy@CFs, PB@CFs, and PB/PPy@CFs. Natural cotton fiber is cellulose I and monoclinic crystal, a = 0.834 nm, b = 1.04 nm, c = 0.789 nm and β = 83.2° [27]. The characteristic diffraction peaks in the XRD spectrum of CFs near 14.9°, 16.3°, and 22.6° corresponded to the (101), ($10\bar{1}$), and (002) crystal planes of cellulose I, respectively. After coating PPy on CFs, no obvious PPy diffraction peak was found, indicating that PPy was coated on the surface of CFs in amorphous form [28]. FeFe(CN)_6_ nanoparticles have face centered cubic (FCC) lattice structure, high crystallinity, and no impurity phase. In the crystal diffraction pattern of PB@CFs, the characteristic diffraction peaks at about 17.5°, 24.7°, 35.4°, and 39.8° corresponded to the (200), (220), (400), and (420) crystal planes, which were consistent with the database of standard card (JCPDS card No. 73-0687) [29], indicating that crystalline FeFe(CN)_6_ nanoparticles had been successfully grown on CFs. The characteristic diffraction peaks of CFs, PPy@CFs, and PB@CFs were all appeared in the diffraction pattern of PB/PPy@CFs, which confirmed the PB/PPy@CFs composite was successfully prepared.

#### 3.2.3. FTIR Analysis

**Figure 5:**
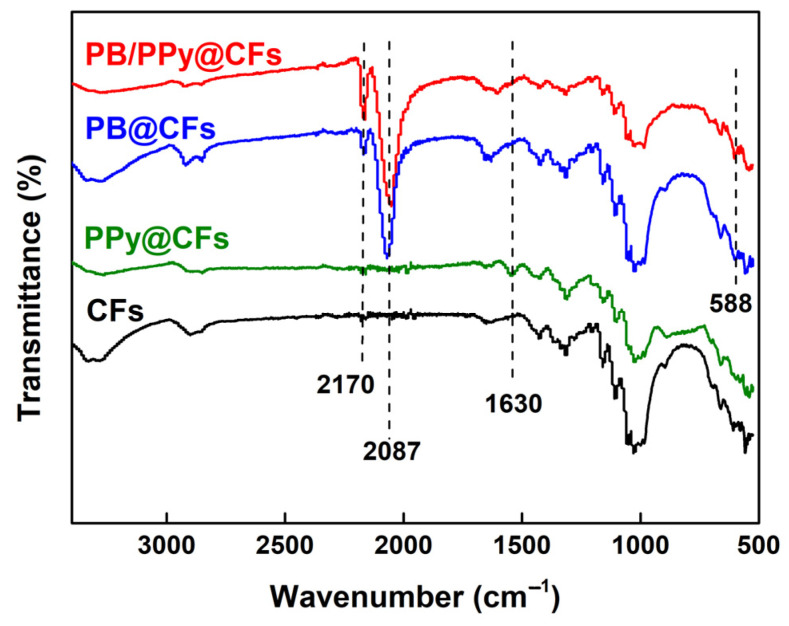
FTIR spectra of CFs, PPy@CFs, PB@CFs, and PB/PPy@CFs.

Figure 5 shows FTIR spectra of CFs, PPy@CFs, PB@CFs, and PB/PPy@CFs. The spectrum of CFs had obvious characteristic peaks of cellulose. The wide peak at about 3300 cm^−1^ belonged to O–H stretching vibration, and the peak at about 2880 cm^−1^ belonged to C–H stretching vibration. The absorption peak at about 1630 cm^−1^ in the spectrum of PPy@CFs was assigned to the N–H stretching vibration of PPy. The strong absorption bands at 2087 cm^−1^ and 2170 cm^−1^ in the spectrum of PB@CFs belonged to C≡N functional groups. The absorption band (650–450 cm^−1^) in the far infrared region was attributed to the Fe–C≡N–Fe bending mode characteristics of FeFe(CN)_6_, in which the peak at 588 cm^−1^ was the result of Fe-C vibration [30]. There were all the characteristic peaks of CFs, PPy@CFs and PB@CFs in the spectrum of PB/PPy@CFs, which proved the successful preparation of PB/PPy@CFs composite.

#### 3.2.4. XPS Analysis

**Figure 6:**
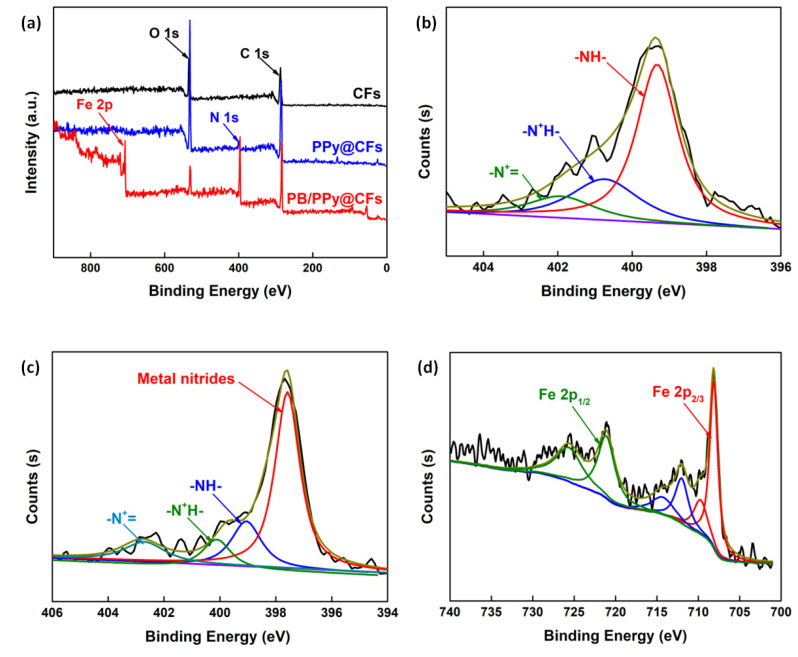
(**a**) XPS wide scan spectra of CFs, PPy@CFs, and PB/PPy@CFs; (**b**) N 1s XPS spectrum of PPy@CFs; N 1s (**c**) and Fe 2p (**d**) XPS spectra of PB/PPy@CFs.

The XPS spectra of CFs, PPy@CFs, and PB/PPy@CFs further provided rich information about the chemical state of the surface elements of the composites. As shown in Figure 6a, C and O peaks appeared in all samples, which were attributed to the rich hydroxyl groups in the carbon skeleton of organic compounds and cellulose matrix. The N element peak represents PPy and FeFe(CN)_6_, and the Fe element peak represents FeFe(CN)_6_. The above characteristic peaks appeared in PB/PPy@CFs, which indicated that both PPy and PB were deposited on the surface of CFs, and PB/PPy@CFs composite was successfully prepared. Figure 6b shows N 1s narrow scan XPS spectrum of PPy@CFs, and the N 1s could be fitted into three peaks of 399.4, 401, and 402.3 eV, corresponding to –NH–, –N^+^H– and –N^+^= bonds, respectively [31]. In Figure 6c, in addition to the three peaks in Figure 6b, there was the characteristic peak of metal nitrides at about 397.65 eV, indicating that metal ions existed in PB/PPy@CFs composite. Figure 6d shows the Fe 2p XPS spectrum of PB/PPy@CFs. The peaks with binding energies of 708.48 eV and 710.24 eV corresponded to Fe 2P_3/2_ orbit, and the peaks with binding energies of 721.38 eV and 724.96 eV corresponded to Fe 2p_1/2_ orbit, indicating Fe^3+^ existed in the PB/PPy@CFs composite. In conclusion, the XPS results clearly showed that the PB/PPy@CFs composite was successfully prepared.

#### 3.2.5. UV-Vis-NIR Spectroscopy Analysis

**Figure 7:**
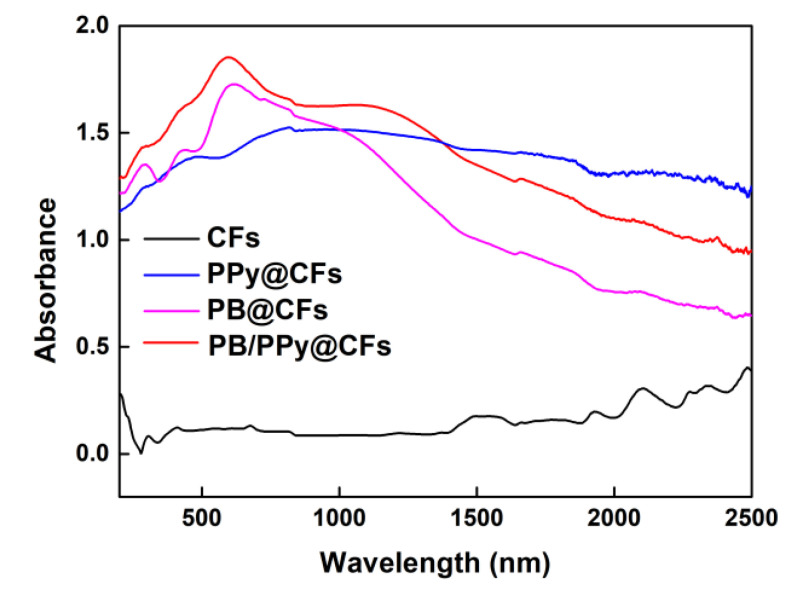
UV-Vis-NIR absorption spectra of CFs, PPy@CFs, PB@CFs, and PB/PPy@CFs in the wavelength range of 200–2500 nm.

The light absorptivity of photothermal conversion materials to solar spectrum is an important factor affecting their water evaporation performance. Figure 7 shows the absorption spectra of CFs, PB@CFs, PPy@CFs, and PB/PPy@CFs. As shown in Figure 7, CFs basically did not absorb sunlight, which is mainly because cellulose itself has no chromophoric group. Both PB@CFs and PPy@CFs had a high solar absorption capacity. PB@CFs exhibited high light absorption, especially in the ultraviolet and visible wavelength range (200–800 nm), which is mainly based on the charge transfer between the metal from Fe^2+^ to Fe^3+^. PPy/CFs had better absorption in the infrared wavelength range (800–2500 nm), which is mainly based on its ability to convert incident photons into heat via nonradiative relaxation and molecular vibrations in their chains. Compared to PB@CFs and PPy@CFs, the composite product (PB/PPy@CFs) containing both PPy and PB absorbed sunlight more effectively in the whole solar spectrum, which makes up for the lack of both PB@CFs and PPy@CFs.

### 3.3. Photothermal Conversion Properties

#### 3.3.1. Wettability

**Figure 8:**
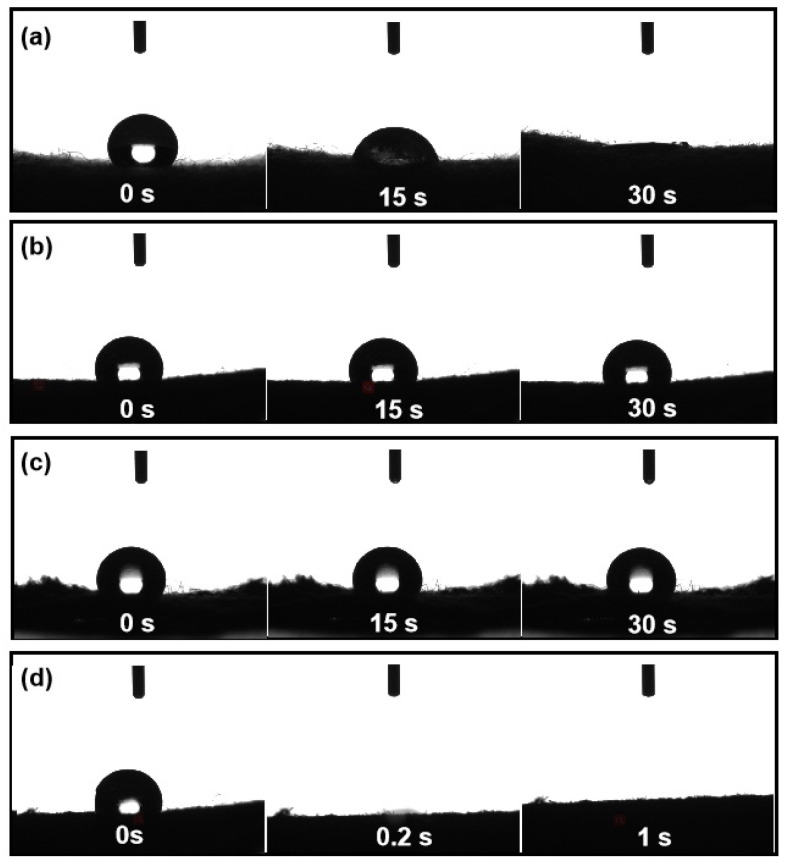
Water contact angle test images of CFs (**a**), PPy@CFs (**b**), PB@CFs (**c**), and PB/PPy@CFs (**d**).

Water evaporation is an interface phenomenon, which is a process that liquid water at the vapor-liquid interface turns into water vapor after converting the absorbed heat energy into kinetic energy. Therefore, whether the photothermal conversion material can form a continuous water supply on the material surface will have an important impact on the sustainability of water vapor in the evaporation process. Figure 8 shows the water contact angle test images of CFs, PPy@CFs, PB@CFs, and PB/PPy@CFs. As observed from Figure 8, 0.5 μL of water droplet was infiltrated into the CFs within 30 s, whereas into the PB/PPy@CFs composite within fraction of a second. However, there was no obvious water droplet infiltration into PB@CFs and PPy@CFs within 30 s. The above results indicated that the PB/PPy@CFs composite had the best hydrophilicity.

**Figure 9:**
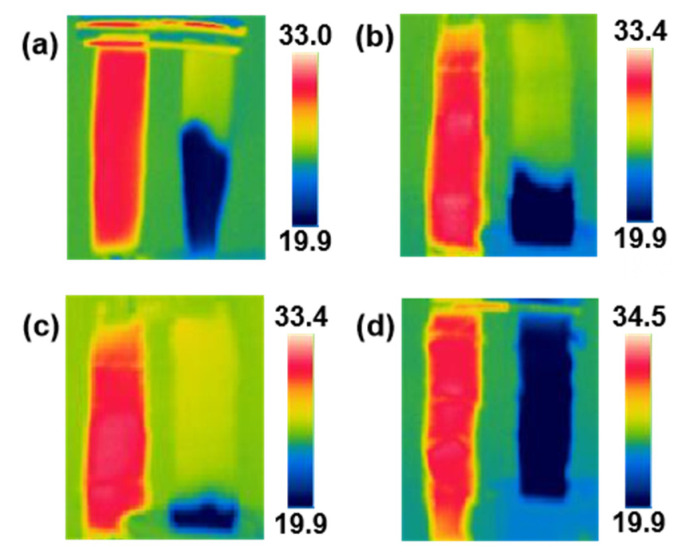
Infrared imaging photos of CFs (**a**), PPy@CFs (**b**), PB@CFs (**c**), and PB/PPy@CFs (**d**) before and after wetting.

Figure 9 shows the infrared imaging photos of CFs, PPy@CFs, PB@CFs, and PB/PPy@CFs before and after wetting taken by the infrared imager. In Figure 9a–d, the samples on the left were placed in the air, and the infrared imaging photos were displayed as red; but the bottom ends of the samples on the right were immersed in water, and the infrared imaging photos were displayed as dark blue. It was observed that the surface temperature of the wet sample was lower than that of the dry sample, which is related to the reduction of the sample surface temperature due to the natural evaporation of water [32]. Meanwhile, the surface temperature distribution of the wetted PB/PPy@CFs sample was uniform, indicating that water was evenly transferred from the bottom to the top, and the PB/PPy@CFs composite had excellent wettability and hydraulic conductivity.

#### 3.3.2. Photothermal Performance

**Figure 10:**
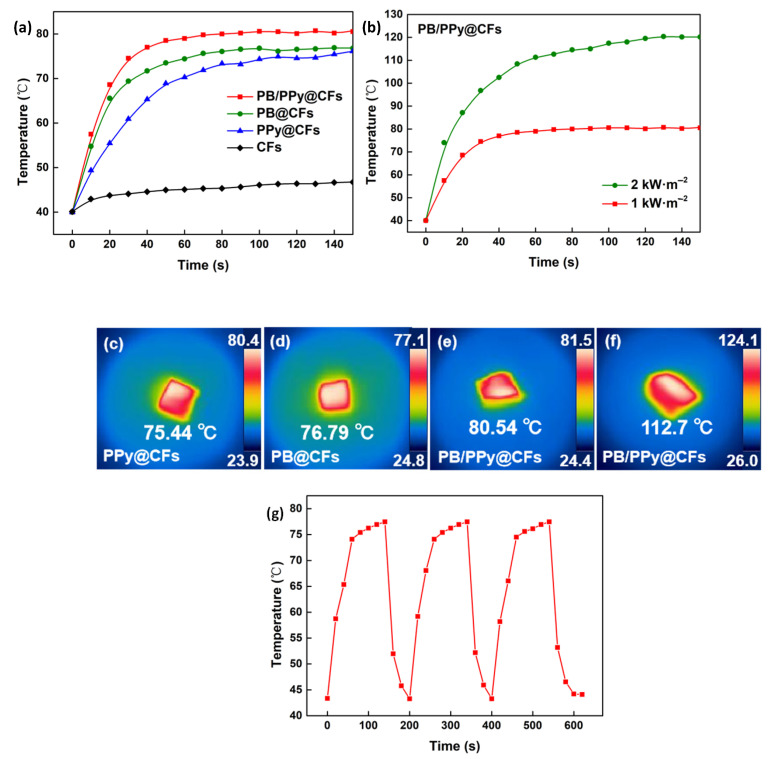
(**a**) Variation of surface temperature of different samples with time at 1 kW·m^−2^; (**b**) Variation of surface temperature of PB/Ppy@CFs with time at 1 kW·m^−2^ and 2 kW·m^−2^; (**c**–**e**) Infrared imaging photos of Ppy@CFs, PB@CFs, and PB/PPy@CFs at 1 kW·m^−2^; (**f**) Infrared imaging photo of PB/PPy@CFs at 2 kW·m^−2^; (**g**) Heating/cooling cycle of PB/PPy@CFs at 1 kW·m^−2^.

The xenon lamp was used to simulate solar radiation to study the photothermal performance of PPy@CFs, PB@CFs, and PB/PPy@CFs composites in the dry state. As shown in Figure 10a, under the illumination of a standard sun (1 kW·m^−2^), PPy@CFs, PB@CFs, and PB/PPy@CFs composites were rapidly heated, but CFs only reached 45.6 °C under 150 s illumination. After 150 s illumination at 1 kW·m^−2^, the surface temperatures of PPy@CFs, PB@CFs, and PB/PPy@CFs increased to 80.4 °C, 77.1 °C, and 81.5 °C, respectively (Figure 10c–e). After two standard sun illumination for 150 s, the surface temperature of PB/PPy@CFs could reach 124.1 °C (Figure 10b,f). The above results indicated that the PB/Ppy@CFs composite had the best photothermal performance. This was because PB nanoparticles were evenly distributed and had strong light absorption capacity. At the same time, Ppy is also a polymer with excellent photothermal performance, which can absorb near-infrared light and convert it into heat energy [33]. As shown in Figure 10g, through repeated heating/cooling cycles (turning on the xenon lamp for 150 s and then turning off for 100 s), three cycles confirmed the stable and fast photothermal conversion ability of PB/Ppy@CFs.

**Figure 11:**
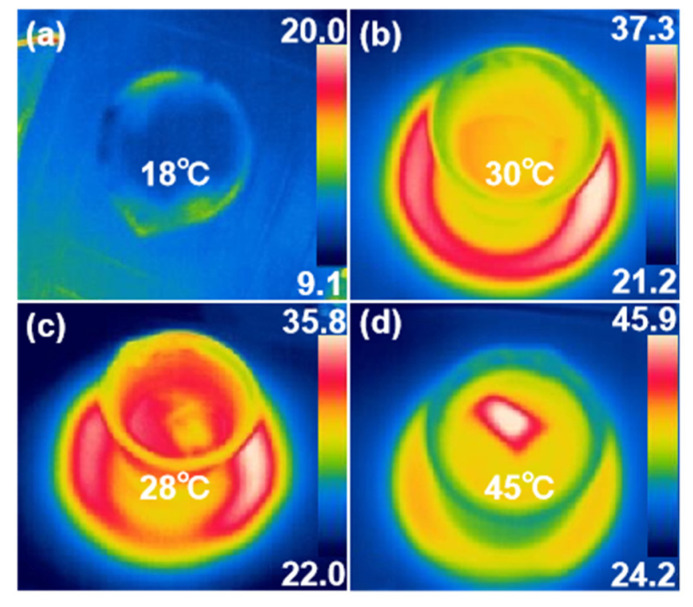
Infrared thermal imaging photos: (**a**) Pure water evaporation system in dark; Pure water evaporation system (**b**), CFs-foam water evaporation system (**c**) and PB/PPy@CFs-foam water evaporation system (**d**) after continuous irradiation for 15 min at 1 kW·m^−2^.

Different from the traditional method of heating the whole water area, the most prominent advantage of solar-driven interface water evaporation is heating interface water. Thus, a local high temperature region is formed at the vapor–liquid interface to realize efficient water evaporation. The surface temperatures of different water evaporation systems were recorded by infrared imager to evaluate the photothermal performance, and the results are shown in Figure 11. The surface temperature of the pure water evaporation system is 20 °C in dark (Figure 11a). After continuous irradiation at 1 kW·m^−2^ optical concentration for 15 min, the surface temperature of pure water evaporation system increased to 37.3 °C (Figure 11b). In the pure water evaporation system, when heating the whole water area, a large number of water bodies absorbed heat, the temperature of water in container increased as a whole and the heat was transferred to the surrounding environment, resulting in serious heat loss, so the surface temperature of water was low. The surface temperature of the CFs-foam water evaporation system was 35.8 °C (Figure 11c), slightly lower than that of pure water evaporation system, which might be caused by the reflection of sunlight on the surface of the pure white CFs. The surface temperature of PB/PPy@CFs-foam water evaporation system was as high as 45.9 °C (Figure 11d), which was mainly due to the excellent light absorption performance of PB for sun light and the ability to convert the absorbed light energy into heat energy.

#### 3.3.3. Water Evaporation Performance

**Figure 12:**
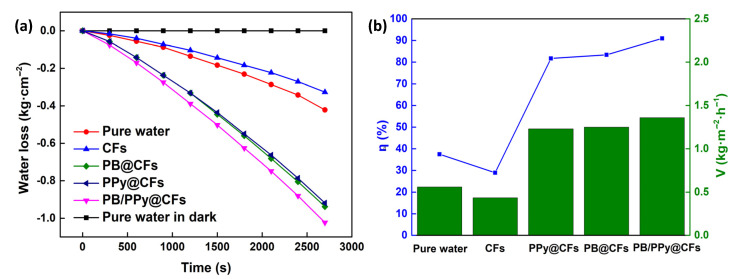
(**a**) Water mass loss of different samples; (**b**) Water evaporation rate and photothermal conversion efficiency.

Figure 12a shows the water mass loss of pure water evaporation system in dark and pure water evaporation system at 1 kW·m^−2^, CFs-foam, PB@CFs-foam, PPy@CFs-foam, and PB/PPy@CFs-foam water evaporation systems at 1 kW·m^−2^. Among them, the water mass loss of pure water evaporation system in dark reflects the natural evaporation of water. This value needs to be subtracted when calculating the water mass loss of different water evaporation systems irradiated with light. After continuous irradiation at an optical concentration of 1 kW·m^−2^ for 45 min, the water mass loss of PB/PPy@CFs-foam water evaporation system caused by evaporation was much higher than that of pure water evaporation system, which was because the water below PB/PPy@CFs was transferred to the surface of PB/PPy@CFs through capillary action to form a local thermal region, which accelerated the evaporation of water. According to Figure 12a, the water evaporation rates were calculated. After continuous irradiation at 1 kW·m^−2^ for 45 min, the water evaporation rates of pure water, CFs, PB@CFs, PPy@CFs, and PB/PPy@CFs systems were 0.56, 0.44, 1.23, 1.25, and 1.36 kg·m^−2^·h^−1^, respectively, and the corresponding photothermal conversion efficiencies were 37.50%, 28.98%, 81.69%, 83.38%, and 90.96%, respectively, as shown in Figure 12b. The water evaporation rate of PB/PPy@CFs was the largest, and the light heat conversion efficiency was the highest, which could realize efficient interface water evaporation.

#### 3.3.4. Stability

**Figure 13:**
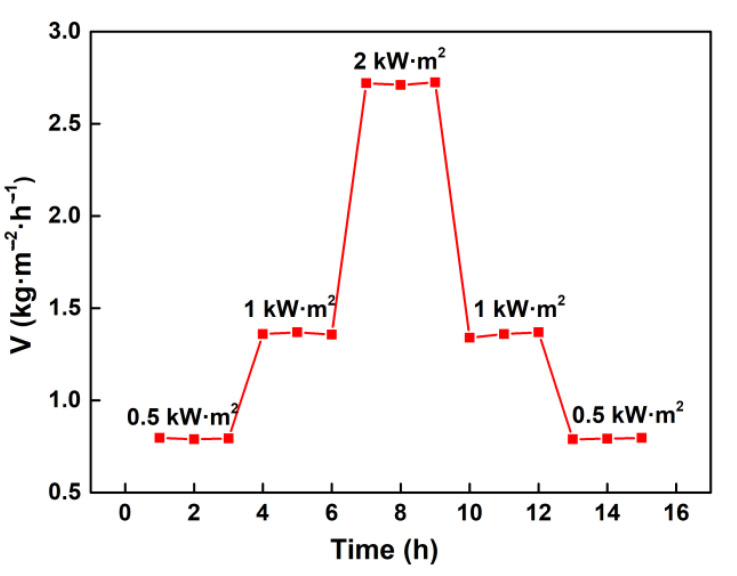
Water evaporation rates of PB/PPy@CFs at different optical concentrations.

The stability of photothermal material is indispensable for practical applications. Good stability is conducive to material reuse and cost saving. This is very necessary for large-scale industrialized production. Figure 13 shows the water evaporation rates of PB/PPy@CFs-foam water evaporation system obtained after continuous irradiation at different optical concentrations. The optical concentration increased first and then decreased. Each optical concentration was irradiated continuously for 2 h. As shown in Figure 13, the greater the optical concentration, the greater the water evaporation rate, indicating that the photothermal conversion material could convert light energy into more heat energy, so as to promote the rapid evaporation of water. Meanwhile, at the same optical concentration, the fluctuation of water evaporation rate was very small, which showed that the photothermal material had good stability.

## 4. Conclusions

The in-situ generated proper amounts of polypyrrole (PPy) doped with phytic acid (PA) as a mediating layer effectively promoted the growth of Prussian blue (PB) on cotton fibers (CFs), and the PB deposition ratio increased from 12.29% (PB@CFs) to 32.4% (PB/PPy@CFs). The growth of PB on PPy@CFs could be completed in 4 h. The PPy coating and PB growth on CFs were confirmed by SEM, XRD, FTIR, XPS, and UV-Vis-NIR spectroscopy. The PB/PPy@CFs composite had excellent light absorption, hydrophilicity, wettability, and photothermal property, and the surface in the dry state could be heated up to 81.5 °C under one sun illumination. The water evaporation rate of PB/PPy@CFs composite as a photothermal conversion material was 1.36 kg·m^−^^2^·h^−^^1^, the corresponding photothermal conversion efficiency increased from 81.69% (PB@CFs) to 90.96% (PB/PPy@CFs) and had good stability.

## Data Availability

The authors confirm that the data supporting the findings of this study is available within the article.
